# Supplementary material for: Detailed molecular and epigenetic characterization of the pig IPEC-J2 and chicken SL-29 cell lines
Source: iScience. 2023 Feb 20;26(3):106252. doi: 10.1016/j.isci.2023.106252 (PMC10018572; doi:10.1016/j.isci.2023.106252)
Supplement: Data S2. Complete homer output for identified motifs in Chicken SL-29, related to Tables 5 and 6 — Homer motif analysis results for histone modifications H3K4me1, H3K4me3, H3K27ac, enhancers, and ATAC-seq of chicken SL-29 cell line. Parameters for possible false positives is as mentioned earlier for S5. [file mmc3.zip › Data_S2/S6/Chicken_SL_29/peak_files_H3K27ac/homerResults.html]

peak\_files\_H3K27ac/ - Homer de novo Motif Results


# Homer *de novo* Motif Results (peak\_files\_H3K27ac/)

Known Motif Enrichment Results  
Gene Ontology Enrichment Results  
If Homer is having trouble matching a motif to a known motif, try copy/pasting the matrix file into
STAMP  
More information on motif finding results: HOMER
| Description of Results
| Tips
  
Total target sequences = 30149  
Total background sequences = 30232  
\* - possible false positive  

|  |  |  |  |  |  |  |  |  |
| --- | --- | --- | --- | --- | --- | --- | --- | --- |
| Rank | Motif | P-value | log P-pvalue | % of Targets | % of Background | STD(Bg STD) | Best Match/Details | Motif File |
| 1 | A C T G G T C A A C T G A C T G A G T C A G T C A G C T C G T A | 1e-86 | -1.987e+02 | 9.38% | 6.41% | 82.4bp (91.9bp) | ZNF711(Zf)/SHSY5Y-ZNF711-ChIP-Seq(GSE20673)/Homer(0.867) More Information \| Similar Motifs Found | motif file (matrix) |
| 2 | T C A G T A G C T A G C C G T A A C G T T A C G C A T G A G T C A T C G C T A G A T G C A T C G T A C G | 1e-78 | -1.818e+02 | 6.05% | 3.80% | 93.2bp (93.9bp) | YY2/MA0748.2/Jaspar(0.946) More Information \| Similar Motifs Found | motif file (matrix) |
| 3 | T A G C A T G C A T G C G A C T T A C G G C T A T C A G C A T G T C A G T C G A | 1e-59 | -1.381e+02 | 13.78% | 10.75% | 83.7bp (91.8bp) | AP-2alpha(AP2)/Hela-AP2alpha-ChIP-Seq(GSE31477)/Homer(0.727) More Information \| Similar Motifs Found | motif file (matrix) |
| 4 | T C G A C T G A G C A T A G C T A T G C A T G C A G C T T A C G | 1e-48 | -1.126e+02 | 59.84% | 55.63% | 85.9bp (88.2bp) | SPDEF(ETS)/VCaP-SPDEF-ChIP-Seq(SRA014231)/Homer(0.790) More Information \| Similar Motifs Found | motif file (matrix) |
| 5 | A T C G T A C G T C G A C T G A T C G A G T A C A G T C A T G C | 1e-46 | -1.065e+02 | 22.25% | 18.94% | 84.3bp (88.8bp) | IRF4(IRF)/GM12878-IRF4-ChIP-Seq(GSE32465)/Homer(0.682) More Information \| Similar Motifs Found | motif file (matrix) |
| 6 | A T C G A T G C A G T C A T C G A C T G C A G T C G A T G T C A | 1e-46 | -1.062e+02 | 36.92% | 33.00% | 85.4bp (94.2bp) | MYB(HTH)/ERMYB-Myb-ChIPSeq(GSE22095)/Homer(0.748) More Information \| Similar Motifs Found | motif file (matrix) |
| 7 | A C G T C G T A A T C G A C T G A T C G A C T G A G T C A C G T | 1e-41 | -9.485e+01 | 18.22% | 15.35% | 84.1bp (87.8bp) | LRF(Zf)/Erythroblasts-ZBTB7A-ChIP-Seq(GSE74977)/Homer(0.709) More Information \| Similar Motifs Found | motif file (matrix) |
| 8 | G A T C G C A T C G T A T A C G C G A T T G C A C T G A C A T G G T C A T C G A T G C A C T G A C T A G | 1e-39 | -9.072e+01 | 0.14% | 0.01% | 82.6bp (81.7bp) | Cdx2(Homeobox)/mES-Cdx2-ChIP-Seq(GSE14586)/Homer(0.639) More Information \| Similar Motifs Found | motif file (matrix) |
| 9 | C A G T A C G T A G T C G C T A C A G T A C T G A T C G C G T A T C A G C G T A T C A G C G A T C A T G | 1e-37 | -8.611e+01 | 0.11% | 0.01% | 85.8bp (8.1bp) | Stat5a/MA1624.1/Jaspar(0.717) More Information \| Similar Motifs Found | motif file (matrix) |
| 10 | T C A G A T C G A T C G A G T C G A T C A G C T T C A G A C T G A T C G G A T C A T G C A G C T A T C G | 1e-37 | -8.605e+01 | 1.58% | 0.83% | 77.0bp (89.6bp) | Zfx/MA0146.2/Jaspar(0.818) More Information \| Similar Motifs Found | motif file (matrix) |
| 11 | T G A C T C G A A T C G G T C A A C T G A T C G T G A C C A T G A T G C A G T C A G C T A G T C A G C T | 1e-34 | -7.914e+01 | 0.10% | 0.00% | 80.6bp (27.8bp) | TFAP2C(var.2)/MA0814.2/Jaspar(0.560) More Information \| Similar Motifs Found | motif file (matrix) |
| 12 | C G A T A T G C A G C T G T A C C G T A C G T A T C G A T A G C A G T C C T G A C G T A C A T G G T A C | 1e-34 | -7.914e+01 | 0.10% | 0.00% | 85.3bp (0.0bp) | POL001.1\_MTE/Jaspar(0.588) More Information \| Similar Motifs Found | motif file (matrix) |
| 13 | C A G T C A G T A G C T T A G C C G A T A T C G A T G C A T G C | 1e-33 | -7.714e+01 | 47.13% | 43.66% | 84.7bp (89.8bp) | E2F1(E2F)/Hela-E2F1-ChIP-Seq(GSE22478)/Homer(0.666) More Information \| Similar Motifs Found | motif file (matrix) |
| 14 | A T G C A C G T A T C G C A G T A C T G C G T A C T G A C T G A | 1e-31 | -7.281e+01 | 35.13% | 31.94% | 84.7bp (88.3bp) | Rbpj1(?)/Panc1-Rbpj1-ChIP-Seq(GSE47459)/Homer(0.712) More Information \| Similar Motifs Found | motif file (matrix) |
| 15 | T G A C G A C T C T G A A C G T T A G C C G T A C A T G T A C G C G T A A C T G G T C A C T G A G T A C | 1e-31 | -7.280e+01 | 0.12% | 0.01% | 71.1bp (32.2bp) | Znf263(Zf)/K562-Znf263-ChIP-Seq(GSE31477)/Homer(0.600) More Information \| Similar Motifs Found | motif file (matrix) |
| 16 | C T G A C G T A T C G A A G T C G C T A A T G C A G T C A T C G | 1e-30 | -7.047e+01 | 42.51% | 39.23% | 85.0bp (91.0bp) | POL010.1\_DCE\_S\_III/Jaspar(0.644) More Information \| Similar Motifs Found | motif file (matrix) |
| 17 | A G C T T A G C G T C A C G T A C G T A T C A G G T A C G T C A C G T A G A T C G C A T A T G C G T A C | 1e-29 | -6.704e+01 | 0.11% | 0.01% | 76.3bp (50.4bp) | TCF7/MA0769.2/Jaspar(0.676) More Information \| Similar Motifs Found | motif file (matrix) |
| 18 | T G A C C T A G A C T G A T G C T A C G A C T G A G C T C G T A C T G A A C T G A C T G A T G C A T G C | 1e-29 | -6.681e+01 | 0.13% | 0.01% | 76.3bp (45.9bp) | E2F6/MA0471.2/Jaspar(0.703) More Information \| Similar Motifs Found | motif file (matrix) |
| 19 | G C A T C T A G A C T G T C A G G T C A T A G C C G T A C T A G T A C G T G A C G A T C G T C A C G T A | 1e-27 | -6.420e+01 | 0.11% | 0.01% | 83.1bp (97.3bp) | PRDM10(Zf)/HEK293-PRDM10.eGFP-ChIP-Seq(Encode)/Homer(0.716) More Information \| Similar Motifs Found | motif file (matrix) |
| 20 | C G T A A T C G T A G C G T A C C G T A T A C G T G C A G C A T A T G C G C A T C A T G A C T G T A G C | 1e-27 | -6.229e+01 | 0.09% | 0.00% | 81.6bp (59.8bp) | ZBTB18/MA0698.1/Jaspar(0.650) More Information \| Similar Motifs Found | motif file (matrix) |
| 21 | A C T G C T A G C G A T C T A G C G T A A C T G C G A T C T A G A G T C A C T G | 1e-26 | -6.008e+01 | 0.83% | 0.39% | 81.1bp (96.1bp) | MSANTD3/MA1523.1/Jaspar(0.755) More Information \| Similar Motifs Found | motif file (matrix) |
| 22 | A C G T A T C G A C G T A C T G G T A C G T C A G T C A A C G T A G T C G T C A A C T G C G A T C G T A | 1e-25 | -5.862e+01 | 0.10% | 0.01% | 85.7bp (53.4bp) | CEBPA/MA0102.4/Jaspar(0.684) More Information \| Similar Motifs Found | motif file (matrix) |
| 23 | C G T A T C A G C T G A C T A G G T A C G C A T C A T G G A C T C A G T C G T A C G A T G T C A G C T A | 1e-24 | -5.587e+01 | 0.10% | 0.01% | 77.1bp (62.2bp) | MSC/MA0665.1/Jaspar(0.695) More Information \| Similar Motifs Found | motif file (matrix) |
| 24 | T C G A T G C A C T G A T A C G C T G A C T A G C T A G T C A G T G C A G A T C G C A T A C G T A T G C | 1e-24 | -5.587e+01 | 0.10% | 0.01% | 75.3bp (74.3bp) | SpiB(ETS)/OCILY3-SPIB-ChIP-Seq(GSE56857)/Homer(0.630) More Information \| Similar Motifs Found | motif file (matrix) |
| 25 | A T C G T G A C C G A T C A T G T A C G T G A C T G C A T A G C T A C G C T G A T A C G T C G A C T A G | 1e-24 | -5.587e+01 | 0.10% | 0.01% | 63.6bp (73.6bp) | HIC1(Zf)/Treg-ZBTB29-ChIP-Seq(GSE99889)/Homer(0.717) More Information \| Similar Motifs Found | motif file (matrix) |
| 26 | C G T A C A T G A C T G G A T C G C A T A T G C G C T A A C G T C A G T A C T G G A T C G C A T A C G T | 1e-24 | -5.587e+01 | 0.10% | 0.01% | 75.7bp (43.5bp) | NR1H4/MA1110.1/Jaspar(0.590) More Information \| Similar Motifs Found | motif file (matrix) |
| 27 | G C T A G T A C G C A T A T C G G T C A C G A T A C T G C T G A G T A C G C T A C G T A C G A T C A G T | 1e-23 | -5.316e+01 | 0.10% | 0.01% | 80.0bp (129.3bp) | PB0165.1\_Sox11\_2/Jaspar(0.653) More Information \| Similar Motifs Found | motif file (matrix) |
| 28 | A G T C A C T G A G T C A C G T A C G T C G T A C G T A C G T A C G T A C G T A C G T A C G T A A C T G | 1e-22 | -5.262e+01 | 0.08% | 0.01% | 75.0bp (39.0bp) | ZNF384/MA1125.1/Jaspar(0.677) More Information \| Similar Motifs Found | motif file (matrix) |
| 29 | G T A C C G A T A G C T G A C T C G A T C G A T C T G A T C A G C T A G C T A G | 1e-22 | -5.121e+01 | 8.04% | 6.60% | 83.8bp (89.0bp) | Hoxd11(Homeobox)/ChickenMSG-Hoxd11.Flag-ChIP-Seq(GSE86088)/Homer(0.681) More Information \| Similar Motifs Found | motif file (matrix) |
| 30 | A T G C A T C G C A T G G T C A A C G T G C T A T G C A A T C G | 1e-22 | -5.116e+01 | 6.03% | 4.78% | 84.3bp (90.7bp) | GSC2/MA0891.1/Jaspar(0.686) More Information \| Similar Motifs Found | motif file (matrix) |
| 31 | A G T C A C T G A G T C A C G T C G T A A G T C G T A C C T A G | 1e-20 | -4.778e+01 | 6.27% | 5.04% | 88.0bp (93.3bp) | OSR1/MA1542.1/Jaspar(0.779) More Information \| Similar Motifs Found | motif file (matrix) |
| 32 | T C G A T C G A T C G A A G T C G C A T T A G C G C T A T C A G G T A C G C A T T C G A C G T A C T A G | 1e-19 | -4.522e+01 | 0.09% | 0.01% | 93.3bp (16.2bp) | Gfi1b/MA0483.1/Jaspar(0.648) More Information \| Similar Motifs Found | motif file (matrix) |
| 33 | A C T G A T C G A C T G C T G A A G C T A C T G C G T A A G T C A C G T A C G T | 1e-17 | -4.095e+01 | 0.30% | 0.11% | 88.3bp (91.6bp) | Nr2e1/MA0676.1/Jaspar(0.692) More Information \| Similar Motifs Found | motif file (matrix) |
| 34 | G T C A A T C G A G T C A C T G A C G T C G T A C G T A A C T G | 1e-16 | -3.866e+01 | 0.55% | 0.26% | 84.7bp (95.4bp) | ETV6/MA0645.1/Jaspar(0.709) More Information \| Similar Motifs Found | motif file (matrix) |
| 35 | A C T G A C G T C G T A C G T A A C T G A C G T A C T G C G T A A C G T A C G T | 1e-16 | -3.784e+01 | 0.09% | 0.01% | 80.2bp (58.5bp) | Pax7(Paired,Homeobox)/Myoblast-Pax7-ChIP-Seq(GSE25064)/Homer(0.788) More Information \| Similar Motifs Found | motif file (matrix) |
| 36 | C G T A A C T G A G T C C G T A A C T G A C T G A C T G A G T C A C T G C G T A C G T A C G T A A C T G | 1e-14 | -3.347e+01 | 0.08% | 0.01% | 75.6bp (73.4bp) | Zic1::Zic2/MA1628.1/Jaspar(0.617) More Information \| Similar Motifs Found | motif file (matrix) |
| 37 | A T C G A G T C A C G T G T A C C T A G G C T A C G A T G A C T | 1e-14 | -3.312e+01 | 2.02% | 1.45% | 80.3bp (88.3bp) | PB0185.1\_Tcf1\_2/Jaspar(0.687) More Information \| Similar Motifs Found | motif file (matrix) |
| 38 | A G T C C G T A A C G T A C G T C G T A C G T A A T C G A C T G A C G T C G T A | 1e-13 | -3.103e+01 | 0.09% | 0.01% | 83.6bp (67.4bp) | NKX6-3/MA1530.1/Jaspar(0.700) More Information \| Similar Motifs Found | motif file (matrix) |
| 39 | C G T A A C T G A G T C A G T C A C G T A C T G A G T C A C T G A C T G A C G T | 1e-12 | -2.811e+01 | 0.06% | 0.01% | 79.9bp (30.7bp) | RUNX2/MA0511.2/Jaspar(0.687) More Information \| Similar Motifs Found | motif file (matrix) |
| 40 | C G T A C G T A A C T G A C G T A G T C A C G T A C T G A C T G A C T G A C G T | 1e-12 | -2.811e+01 | 0.06% | 0.01% | 83.0bp (45.5bp) | PB0060.1\_Smad3\_1/Jaspar(0.707) More Information \| Similar Motifs Found | motif file (matrix) |
| 41 \* | A G T C A C T G A T G C A C G T A C G T C G T A A G T C C G T A A C T G A G T C | 1e-11 | -2.721e+01 | 0.07% | 0.01% | 76.5bp (0.0bp) | PH0158.1\_Rhox11\_2/Jaspar(0.713) More Information \| Similar Motifs Found | motif file (matrix) |
| 42 \* | A C T G A C G T A C G T A G T C A C G T A C G T A C T G A C G T C G T A A C G T | 1e-10 | -2.325e+01 | 0.07% | 0.01% | 88.5bp (61.8bp) | PB0122.1\_Foxk1\_2/Jaspar(0.653) More Information \| Similar Motifs Found | motif file (matrix) |
| 43 \* | A C G T A C G T A C G T A G T C A C T G C G T A A G T C A C T G | 1e-8 | -2.011e+01 | 0.07% | 0.01% | 101.7bp (61.1bp) | NFAT5/MA0606.1/Jaspar(0.665) More Information \| Similar Motifs Found | motif file (matrix) |
| 44 \* | A C T G C G T A C G T A C G T A C G T A A C G T C G T A A C T G A C T G A G T C | 1e-4 | -1.098e+01 | 0.06% | 0.02% | 92.2bp (89.0bp) | Mef2a(MADS)/HL1-Mef2a.biotin-ChIP-Seq(GSE21529)/Homer(0.732) More Information \| Similar Motifs Found | motif file (matrix) |
